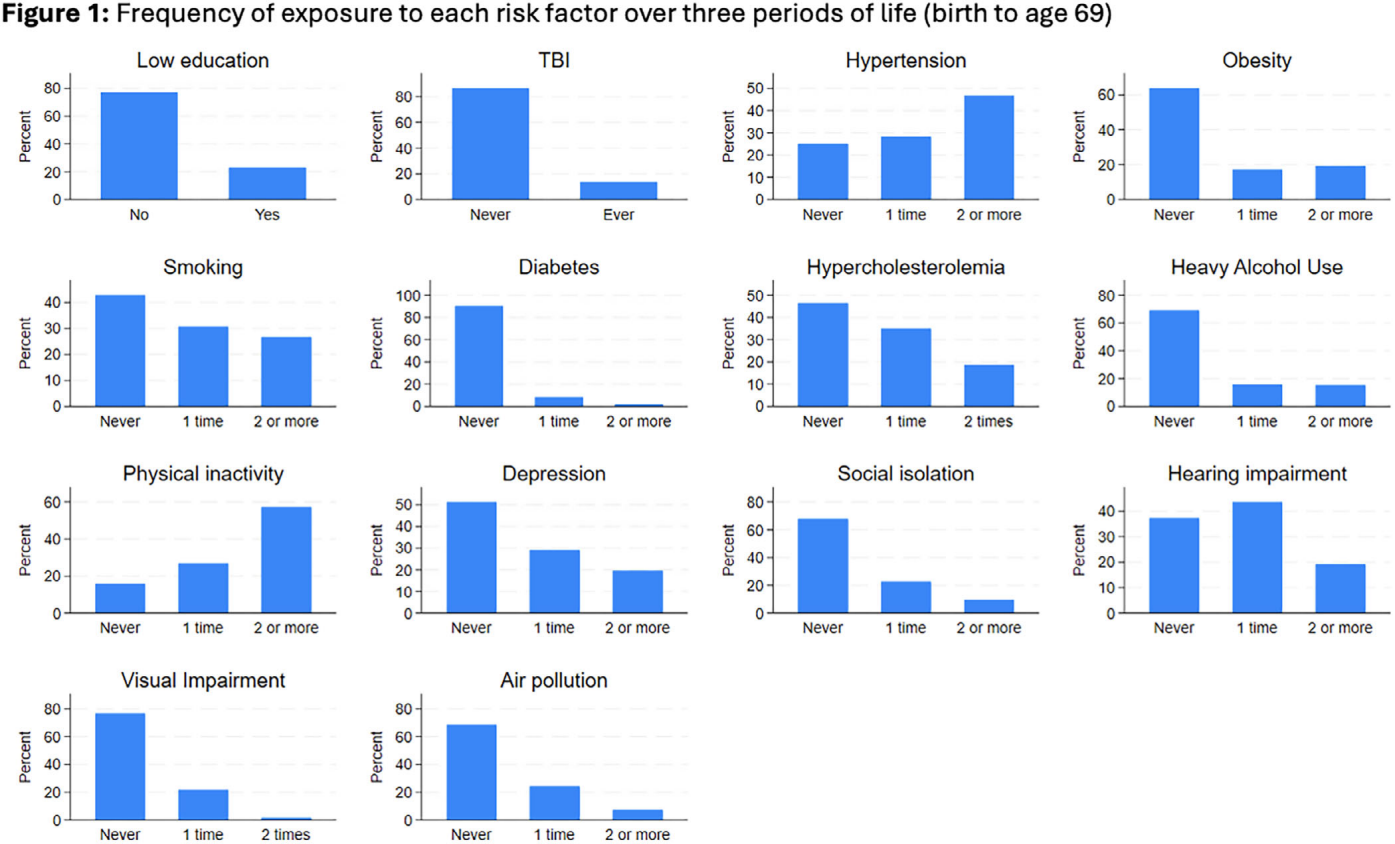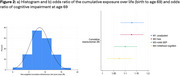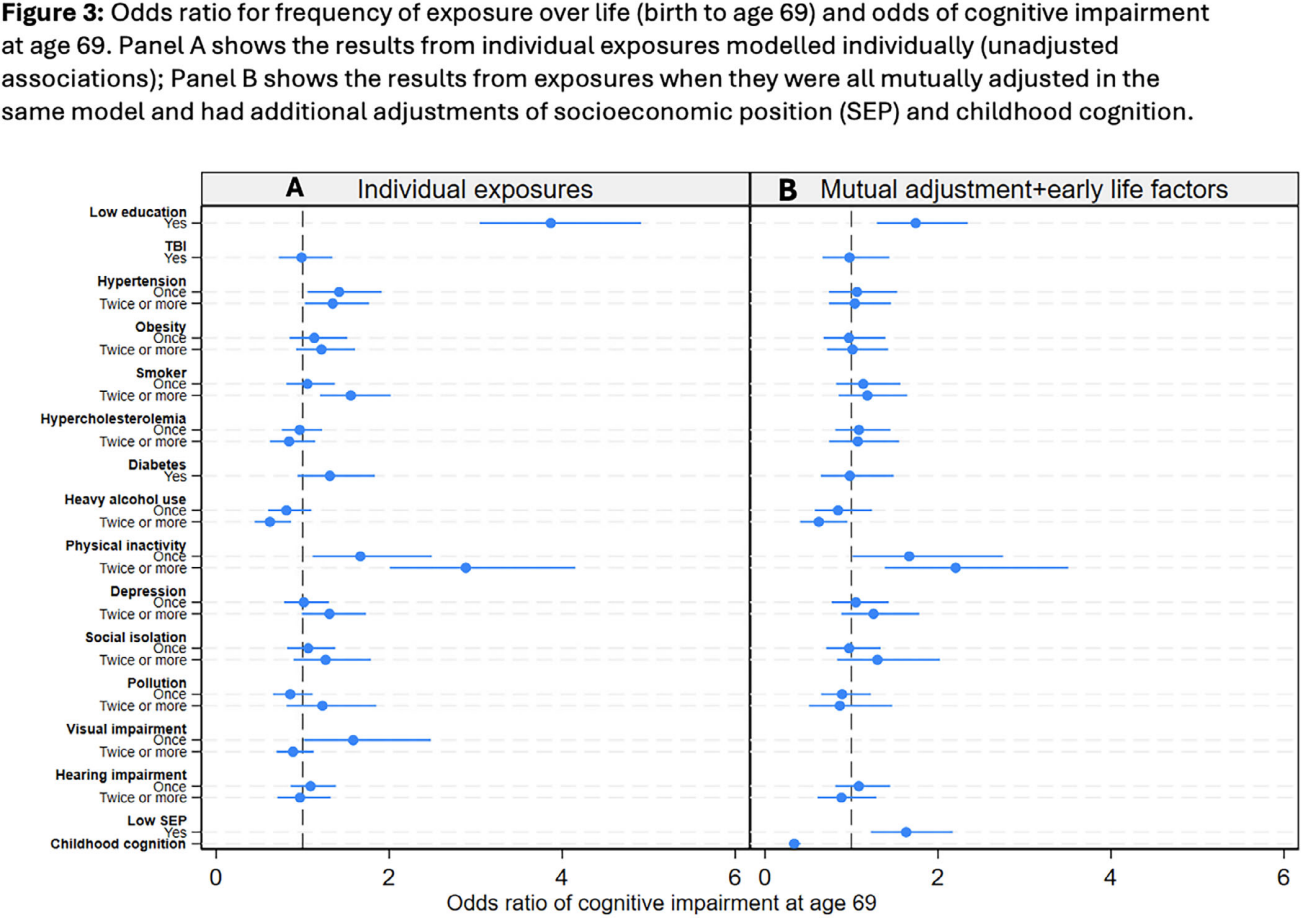# Lifetime exposure to modifiable risks and later‐life cognition impairment: the British 1946 birth study

**DOI:** 10.1002/alz70860_098368

**Published:** 2025-12-23

**Authors:** Sarah‐Naomi James, Louisa P Needham, Jennifer M Nicholas, Marcus Richards, Jonathan M Schott

**Affiliations:** ^1^ University College London, London, England, United Kingdom; ^2^ Department of Medical Statistics, London School of Hygiene and Tropical Medicine, London, United Kingdom

## Abstract

**Background:**

Using an age‐homogenous population‐based sample studied since birth with prospective ‐data collected across the life course, we examined the cumulative and individual relationships between modifiable risks and later‐life cognitive impairment. We examine the role of childhood cognition and socioeconomic position (SEP) in these relationships.

**Method:**

Lifetime exposure to fourteen modifiable dementia risks from the 2024 Lancet commission were derived in the 1946 British Birth Cohort (Table 1) (*n* = 1239, 52% female). Exposures (yes/no) were categorised into three epochs: early‐ [≤36yrs], mid‐ [43‐53yrs], and later‐adulthood [60‐69yrs]); categorised into (0) never exposed, (1) exposed once, (2) exposed ≥2; and summed (max=28). 25% of individuals had cognitive impairment based on a validated threshold (≤88/100) on the Addenbrooke's Cognitive Examination (ACE‐III) administered at 69yrs. Logistic regression models were used to assess relationships between cognitive impairment and exposures.

**Result:**

Participants on average were exposed to 6 (43%) exposures (range:0‐12) (Figure 1). Greater risk exposure (either exposure across another life course period, or to an additional exposure) was associated with cognitive impairment at 69yrs (OR=1.13 [95% CI:1.08‐1.19], *p* <0.01) (Figure 2). This relationship was attenuated by 50% with adjustment for sex, childhood cognition and SEP, but remained robust (OR=1.07 [95 CI: 1.02‐1.14], *p* <0.01).

In individual models, low education, hypertension, long‐term smoking, physical inactivity, long‐term depression and visual impairment were associated with cognitive impairment; alcohol use was linked to a *decreased* risk of cognitive impairment (Figure 3a). However, in mutual adjustment models including childhood cognition and SEP, the only exposures that remained robust were low education and physical inactivity, alongside childhood cognition and SEP (Figure 3b).

**Conclusion:**

While several exposures were associated with cognitive impairment at 69yrs, mostly in a dose‐response manner, the only robust independent relationships were for education and physical inactivity. Baseline cognitive function and social class are also important prediction exposures for later‐life cognitive impairment. These findings emphasise the importance of promoting education, physical activity and improving earlier life socioeconomic status in dementia prevention strategies and demonstrate the potential of longitudinal cohort studies for exploring viable modifiable risks.